# Perspectives on Medical Education: three changes in our guidelines to make authors’ and reviewers’ lives easier

**DOI:** 10.1007/s40037-020-00563-7

**Published:** 2020-02-05

**Authors:** Lauren A. Maggio, Erik W. Driessen

**Affiliations:** 1 Department of Medicine, Uniformed Services University of the Health Sciences, Bethesda, MD USA; 2 Faculty of Health, Medicine and Life Science, Maastricht University, Maastricht, The Netherlands

In 2019, researchers reported that authors spend 52 hours per year formatting manuscripts for submission [1]. At the aggregate level, this burden has been estimated to be over 1.5 million researcher hours annually [2]. On the other side of the manuscript process, researchers estimate that peer reviewers dedicate over 22 million hours (average 5 hours per manuscript) per year to reviewing article submissions [3]. Bottom line: This is a lot of time and effort! Moreover, a large part of this time and effort is unnecessary and frustrating: every resubmission after a rejection requires a new round of reviews; and differences in journal manuscript requirements demand adaptations of your manuscript for every resubmission.

With this in mind at *Perspectives on Medical Education*, we critically reviewed the journal’s policies and procedures. We also analyzed the formatting reasons that cause manuscripts to be returned to authors prior to peer review; a frustrating and time consuming process for all involved. Our intent was to identify, and where possible change, suboptimal policies and procedures to create a more author- and reviewer-friendly experience. Below we announce three changes effective immediately.

## Take two

*Perspectives on Medical Education* now offers a Take Two option, which gives authors a second chance to submit revised manuscripts previously reviewed and rejected from other journals. This approach, based on the ‘Fast Track’ option in use at *Advances in Health Sciences Education *[4], will take into consideration the authors’ revision of their manuscript based on earlier peer reviewer feedback. These submissions will be handled by our team of associate editors to hopefully save time. Additionally, we hope that by taking advantage of the previous reviewers’ time and effort we can cut back on the overall burden on peer reviewers.

Authors who intend to submit to a Take Two manuscript should first email Lieda Meester (lieda.meester@bsl.nl) in the editorial office and provide the following attachments to their message:

- A copy of the previous reviews with the name of the journal
- A table that clearly describes how you addressed each comment and where the change can be found in the revised manuscript
  - If you disagree with a reviewer comment, please describe your rationale in the table
- A clean version of the revised manuscript
- The revised manuscript with track changes indicating all changes

## Single-blind peer review

Authors no longer need to remove identifying elements (e.g., author institution, names, etc.) from their manuscripts upon initial submission. In our analysis, we identified that multiple manuscripts were passed back to authors due to incomplete blinding. Additionally, we found that efforts to remove identifying details often contorted sentences disrupting the reviewer’s read of the manuscript. Lastly, we identified that this created an additional step at copy editing during which the authors needed to add back identifying details.

With this change *Perspectives on Medical Education* transitions from double-blind to a single-blind peer review, such that reviewers will be aware of the identity of the authors, but the peer reviewers’ identities will be unknown to the authors. In making this decision, we reviewed the evidence on blinding in peer review [5–7], which provides a mixed picture of the situation. We will monitor this change for any unexpected implications.

## Format-free tables and figures

When initially submitting to *Perspectives on Medical Education*, authors may now include tables and figures, both in full color or in black and white, directly in their manuscript file. At this point in the process, there is no need to upload these files separately or in a particular format. We encourage authors to position tables and figures in their manuscript where they feel they best represent their work.

If authors are invited to revise and resubmit their manuscript following peer review, at that point they are required to submit tables and figures as separate files and formatted to the journal’s specifications.

We believe this new policy for tables and figures will unburden authors and reduce pass backs prior to review. Plus, we hope it will facilitate peer reviewers in evaluating the manuscript. Reviewers will no longer need to scroll to the end of a manuscript to view tables/figures, but will be able to engage with them as the authors intended.

## Looking ahead

We are excited to enact these changes in our author guidelines, but we see them as just our first few steps. We plan to keep monitoring and studying our own processes and to keep moving towards a more author- and reviewer-friendly publishing process.
